# Transition from Paediatric to Adult Care in Congenital Heart Disease: A Call for Action

**DOI:** 10.3390/jcm14248869

**Published:** 2025-12-15

**Authors:** Fabiola Boccuto, Rosaria Barracano, Giulia Guglielmi, Anamaria Mihailescu, Martina Avesani, Elettra Pomiato, Pierfrancesco Montanaro, Gabriele De Palma, Berardo Sarubbi, Antonella Bruna Cutrì, Jolanda Sabatino, Massimo Chessa, Gianfranco Butera, Claudia Montanaro

**Affiliations:** 1 Division of Cardiology, University Hospital Renato Dulbecco, 88100 Catanzaro, Italy; fabi.boccuto@gmail.com; 2 Adult Congenital Heart Disease Centre, AORN Ospedale dei Colli, Monaldi Hospital, 80131 Naples, Italy; rosariabarracano@libero.it (R.B.); depalma.gabriele97@gmail.com (G.D.P.); berardo.sarubbi@ospedalideicolli.it (B.S.); 3 Pediatric and Adult Congenital Heart Centre, IRCCS Policlinico San Donato, 20097 Milan, Italy; giulia.guglielmi@grupposandonato.it (G.G.); massimo.chessa@grupposandonato.it (M.C.); 4 Emergency Institute for Cardiovascular Diseases Professor Dr. C.C. Iliescu, 022328 Bucharest, Romania; mihailescuanamaria92@gmail.com; 5 Division of Paediatric Cardiology, Department of Women’s and Children’s Health, University Hospital Padua, 35128 Padua, Italy; martina.avesani@aopd.veneto.it (M.A.); elettra.pomiato@aopd.veneto.it (E.P.); 6 Paediatric and Neonatology, Ospedale Santa Maria delle Croci, 48121 Ravenna, Italy; pierfrancescomontanaro@gmail.com; 7 Department of Cardiology, University of Campania Luigi Vanvitelli, 80138 Naples, Italy; 8 Adult Congenital Heart Disease Unit, Bambino Gesù Hospital, Research Institute, 00165 Rome, Italy; antonellabruna.cutri@opbg.net (A.B.C.); gianfranco.butera@opbg.net (G.B.); 9 Department of Experimental and Clinical Medicine, Magna Graecia University, 88100 Catanzaro, Italy; sabatino@unicz.it; 10 School of Medicine, Vita-Salute San Raffaele University, 20132 Milan, Italy; 11 National Heart and Lung Institute, Imperial College London, London SW3 6NP, UK

**Keywords:** congenital heart disease (CHD), transition, adolescence, education, self-management, prevention

## Abstract

**Background:** Transition from paediatric to adult care in congenital heart disease (CHD) represents a pivotal and vulnerable phase that critically influences long-term survival, morbidity, and quality of life. Advances in paediatric cardiology and surgery have generated a rapidly growing population of adults with congenital heart disease who exhibit complex, lifelong, and multidisciplinary needs. However, survival does not equate to cure, and discontinuity of care during adolescence remains a major predictor of adverse outcomes. Despite widespread recognition of their importance, transition programmes are heterogeneous worldwide, and standardised, evidence-based protocols are missing. **Objective:** This review calls for action acknowledging the urgent need for structured and standardised transition programmes in CHD care, integrating the key elements that should be addressed in any programme to optimise outcomes. **Content:** Transition should be understood as a multidisciplinary, longitudinal process integrating medical management, patient and family education, psychological preparation, and societal inclusion. Core domains include tailored physical activity, nutritional counselling, cardiovascular risk factor management, infective endocarditis prevention, reproductive health, psychosocial support, and engagement of primary care providers, educators, and employers. Evidence demonstrates that structured transition programmes enhance health literacy, adherence, and self-management, while reducing loss to follow-up. The active involvement of primary care providers, psychologists, educators, and employers is essential to sustain holistic and equitable care. **Conclusions:** Transition should be reframed as an essential, lifelong component of CHD care. The development and implementation of standardised, multidisciplinary, evidence-based transition protocols are urgently required to ensure continuity, empower patients, and optimise long-term clinical and psychosocial outcomes for adults with CHD.

## 1. Congenital Heart Disease: A Growing Adult Population

Transition from paediatric to adult care in CHD represents a vulnerable phase that can influence long-term survival and quality of life [1].

Advances in paediatric cardiology and surgery have led to a rapidly expanding population of Adult Congenital Heart Disease (ACHD) patients with complex and multidisciplinary needs [2].

However, survival is not equivalent to cure: these individuals face ongoing challenges [3], ranging from arrhythmias and heart failure to pregnancy-related risks, lifestyle vulnerabilities, and psychosocial instability. Discontinuity of care during adolescence remains one of the strongest predictors of adverse outcomes, yet transition programmes worldwide remain inconsistent in timing, structure, and multidisciplinary involvement [4].

This review calls for a paradigm shift: transition should be viewed as a longitudinal, developmentally appropriate process rather than a single transfer event. It must integrate medical management with psychological readiness, lifestyle education, family engagement, and societal inclusion (school, employers, primary care).

In this paper, we explore the pillars of effective transition and propose key reflection points and a structured approach for managing the emerging challenges of adults with CHD—from nutrition to sexual health, from infective endocarditis prevention to pregnancy counselling—with the aim of empowering patients to become informed, autonomous adults without losing cardiologic protection.

### Literature Search Strategy

This narrative review is based on a non-systematic literature search. Relevant studies were identified through PubMed, Scopus, and Google Scholar using combinations of the following keywords: congenital heart disease, transition, adolescents, adult congenital heart disease, education, lifestyle, infective endocarditis prevention, physical activity, and psychosocial support. No formal language restrictions were applied, although the search primarily focused on English-language publications. Additional references were retrieved from citation tracking of key articles. This approach aimed to provide a comprehensive overview of current evidence while acknowledging the narrative nature of the review.

## 2. The Need for Continuity and Integration in Care

Treatment and follow-up of children with CHD are managed by paediatric cardiologists, but transfer to an age-appropriate setting becomes essential as patients grow older. Transfer refers to the formal handover of care, whereas transition describes the gradual medical and psychosocial preparation for adult care [1]. Loss to follow-up frequently occurs during this phase and is associated with adverse outcomes [5,6], with lapses of ≥2 years tripling the risk of urgent cardiac intervention after returning to ACHD care [7]. Structured transition programmes are therefore crucial.

Although evidence remains limited, available trials show that such programmes improve self-management, health behaviours, and disease knowledge, with benefits sustained over time [8,9]. They also help reduce excessive parental involvement and promote healthier self-identity [10]. As CHD patients age, they increasingly face acquired cardiovascular risks such as ASCVD; traditional risk factors—including hypertension, obesity, diabetes, dyslipidaemia, and smoking—further amplify vulnerability and should be addressed early, ideally with family involvement [11,12].

Transition must also address reproductive health, improving awareness of fertility, pregnancy risks, medications, and contraception [13]. Psychological vulnerability is common: CHD patients have markedly higher rates of mental health disorders [14], making psychological support essential for coping, adherence, and long-term outcomes, although only a minority of centres provide integrated services [15,16,17].

Several models exist, including joint clinics, paediatrician-in-adult-care, and introductory approaches, but these often amount to record transfer rather than true transition [1]. The transition coordinator model—offering individualised education and psychosocial support—is considered the most effective [1]. Nurse-led, hybrid, and multidisciplinary clinics further improve health perception, life satisfaction, and psychological well-being [9,18,19].

Despite increasing awareness, European transition practices remain highly variable [17]. A coordinated, evidence-based, and multidisciplinary approach is urgently needed across Europe.

## 3. Patient and Context Education

Transition from paediatric to adult care in CHD is complex and often marked by interruptions in specialised follow-up, which worsen outcomes [1,20]. Engaging patients, families, schools, and employers is therefore essential. A central goal is patient empowerment—improving understanding of CHD and building coping skills and autonomy—while parental support remains fundamental [21]. However, many parents struggle to foster independence [22], leading to stress and overprotective behaviours [23]. Family-centred programmes should therefore guide parents toward a more supportive, consultative role [24].

General practitioners also play a key part in ensuring continuity and preventing loss to follow-up or disease progression [25], particularly within shared-care models linking specialised and community care [26]. Yet, many GPs remain insufficiently informed about CHD management and ACHD services [27,28], highlighting the need for targeted education.

School participation is frequently impaired in CHD, with reduced attendance, need for special support, and lower academic achievement [29]. Adolescents face bullying, discrimination, and difficulties with physical education [30], despite education being a major predictor of later employment success [31]. Schools, employers, and communities should therefore be actively involved. Peer support is particularly valuable [30], and patients often request opportunities to meet others with similar experiences; collaborations with youth ambassadors, patient organisations, and dedicated camps can help [1].

Educational support is often limited by poor intersectoral coordination and inadequate resources [32]. Effective strategies include collaborative planning with healthcare professionals (ideally a school nurse), individualised education plans, positive classroom environments, flexible schedules, and remote learning options to maintain continuity during illness [33]. Employment interventions for complex CHD should emphasise workplace adaptation, flexible hours, fatigue management, and psychosocial support [34].

A multicentre study (>900 patients) showed that 42% experienced gaps in cardiology care, which are linked to urgent procedures and undertreatment [4]. Risk factors include limited patient understanding of their condition, insurance issues, scarce specialist centres, and negative experiences during adult care transitions [35]. By transition age, patients should understand their anatomy, prior surgeries, medications, and the need for regular follow-up [36].

Communication challenges persist due to limited consultation time, variability in clinicians’ expertise, and adolescents’ differing preferences [37]. Some avoid face-to-face discussions and need encouragement to engage, while being guided away from unreliable online sources [1]. Neurocognitive deficits—affecting verbal skills, attention, or working memory—may hinder comprehension, whereas visual memory is often preserved, supporting the use of visual educational tools [1,24].

### Core Elements of a Structured Transition Framework

A comprehensive transition framework for CHD should systematically include the following core components:

- Early initiation: Begin transition planning in early adolescence with progressive goal setting.
- Individualised education: Age-appropriate information on the cardiac condition, treatment, and long-term expectations.
- Development of self-management skills: Encouraging autonomy in medication adherence, symptom recognition, and appointment scheduling.
- Family involvement: Gradual shift from parental control to a supportive, consultative role.
- Integration of primary care: Inclusion of general practitioners within a shared-care model to enhance continuity.
- Psychological assessment and support: Screening for anxiety, depression, and coping difficulties and providing early interventions.
- Lifestyle counselling: Guidance on physical activity, nutrition, smoking cessation, alcohol use, and cardiovascular risk prevention.
- Reproductive health education: Counselling on contraception, pregnancy risks, and preconception planning.
- Structured communication between paediatric and adult teams: Formalised transfer protocols and documentation of transition readiness.
- Continuity of follow-up: Strategies to prevent lapses in care and facilitate consistent engagement with adult CHD services.

In conclusion, these core elements collectively provide a structured, evidence-informed framework that supports a safe, coordinated, and developmentally appropriate transition to adult CHD care.

## 4. Management of Lifestyle and Specific Clinical Issues

### 4.1. Lifestyle and Cardiometabolic Risk Management in ACHD

#### 4.1.1. Nutrition, Body Weight, and Drug–Food Interactions

Healthy nutrition is essential for a healthy life and represents a key element in the prevention of cardiovascular disease. Patients with CHD are predisposed to nutritional disorders such as obesity, which places the growing ACHD population at higher risk for acquired cardiovascular disease.

Evidence has shown that metabolic syndrome (and obesity) is more prevalent in ACHD than in the general population, and its prevalence increases with age [38].

Considering this information, a broader assessment of health behaviours and healthy habits should be implemented beginning in childhood and maintained into adulthood.

The Mediterranean diet is one of the most studied nutritional regimens for cardiovascular disease prevention; it encourages the intake of vegetables, whole grains, fruits, fish and seafood, eggs, dairy products, and unsaturated fatty acids (e.g., olive oil). Although observational studies and clinical trials have demonstrated positive results for cardiovascular prevention with the Mediterranean diet, evidence concerning CHD is scarce, and low adherence has been observed in this patient population [39].

Drug–food interactions are well known and can alter the effectiveness of medications. A notable example is the interaction between warfarin and vitamin K-rich foods (green leafy vegetables, egg yolks, liver), which can affect warfarin efficacy; excessive intake should therefore be avoided to prevent significant INR fluctuations. Grapefruit, pomegranate, cranberries, and soy products should also be avoided due to their potential impact on INR.

Nutritional counselling should be part of ACHD follow-up, as nutritional deficiency is independently associated with an increased risk of major cardiovascular events in ACHD patients. Abnormal calcium metabolism is well known in this population, and vitamin D deficiency is common and should be appropriately supplemented [40].

Iron deficiency is frequent, especially in cyanotic patients, and may affect quality of life and functional class; it is also a negative prognostic marker [41].

While moderate alcohol consumption is allowed for adults with CHD following general population recommendations, it should be strongly discouraged in patients with univentricular physiology due to its potential impact on liver function.

A normal body mass index should be promoted by adopting a low-fat, low-carbohydrate diet to reduce risks associated with metabolic syndrome.

#### 4.1.2. Tobacco Use

Smoking continues to be an important cardiovascular risk factor, and its negative long-term effects are well demonstrated. Cigarette smoke contains over 4000 compounds, but carbon monoxide, reactive oxygen species, and nicotine are primarily responsible for smoking-induced cardiovascular disease (endothelial dysfunction, oxidative stress, and inflammation). Endothelial dysfunction due to smoking may contribute to the development of atherosclerosis [42].

The ACHD population has increased over recent decades due to advances in percutaneous and surgical procedures. As the cardiovascular system of these patients is already affected, smoking may accelerate the progression of atherosclerosis.

In patients with congenital defects involving the aorta and coronary arteries, or in those who have undergone coronary artery reimplantation (e.g., arterial switch operation or Ross procedure), smoking may have significant implications [43].

Smoking is also a risk factor for pulmonary disease, such as chronic obstructive pulmonary disease (COPD). It provokes chronic airway inflammation and reduces exercise tolerance in a population whose cardiopulmonary function and exercise capacity are already compromised [44].

A meta-analysis showed that 1 in 8 (12%) ACHD patients are smokers—a lower prevalence compared with the general population. This difference may be explained by frequent medical appointments that increase awareness of ASCVD risk factors [45].

Adolescence represents a critical period for initiating targeted smoking cessation programmes and should be considered a priority [46]. A meta-analysis showed that family-based interventions (including parents) reduce the overall risk of smoking onset by 24% in young adults [47].

#### 4.1.3. Alcohol Use

Young individuals with CHD, moving into adulthood, are not only navigating complex medical follow-up but are also encountering new freedoms and social pressures. This developmental period is characterised by a strong desire for autonomy and inclusion among peers, which may lead some to experimentation with substances such as alcohol. For patients with congenital heart disease, this exploration carries both medical and psychosocial risks that are often underestimated or insufficiently addressed.

Alcohol interacts with many commonly prescribed cardiac medications. It can alter drug metabolism by inducing or inhibiting hepatic enzymes, thereby modifying the pharmacokinetics and pharmacodynamics of treatments such as anticoagulants, antiarrhythmics, beta-blockers, and diuretics. These interactions may reduce therapeutic efficacy or increase the risk of adverse effects. Moreover, in adults or young adults with CHD who have residual ventricular dysfunction, hepatic congestion, or impaired renal function, even moderate alcohol intake can further stress already compromised organs, potentially leading to clinical decompensation.

In addition, altered haemodynamic status secondary to cardiac disease results in lower tolerance to alcohol compared with age-matched peers, elevating the risk of alcohol-related complications, including arrhythmias, hypotension, and bleeding, especially in patients receiving anticoagulation therapy or those with mechanical valves.

One of the critical issues is that, during paediatric care, alcohol use is rarely discussed in detail. Conversations about substance use are often postponed or considered less urgent compared with anatomical or surgical concerns (such as arrhythmia surveillance or planning re-interventions), unintentionally neglecting important aspects of lifestyle counselling. As a result, many adolescents enter adult care without having received structured, age-appropriate guidance on this topic. For young adults, this may create a gap in care in which they feel unprepared to navigate real-life choices regarding alcohol, sometimes perceiving restrictions as barriers to social participation.

To address this, transition programmes must adopt a proactive and structured approach to alcohol education [48].

The safety threshold for alcohol use is not fixed; it may shift over time based on changes in ventricular function, the development of arrhythmias, or the progression of organ dysfunction. Periodic reassessment and re-counselling are therefore essential components of long-term care planning.

Although no major guidelines currently establish explicit alcohol consumption thresholds specific to ACHD patients, the 2020 ESC Guidelines for the Management of Adult Congenital Heart Disease clearly state that lifestyle factors, including alcohol use, should be routinely addressed in adult congenital care [16].

In conclusion, managing alcohol use during the transition period is not simply a matter of delivering warnings; it requires a structured, personalised, and evolving educational strategy. Empowering young adults to make informed decisions will not only reduce risk but also foster a sense of agency and responsibility that supports long-term health outcomes.

#### 4.1.4. Classical Cardiovascular Risk Factors

As patients with congenital heart disease reach adulthood, their medical journey becomes increasingly shaped not only by their underlying structural defects but also by the gradual emergence of acquired cardiovascular risk factors. Conditions such as hypertension, dyslipidaemia, insulin resistance, and type 2 diabetes become more prevalent with age and interact in potentially dangerous ways with the pre-existing congenital substrate, or they may even develop early as side effects of the disease.

Traditional risk factors may accelerate vascular ageing and atherosclerosis, worsening afterload or preload conditions, particularly in patients with residual valvular defects, outflow tract obstructions, or single-ventricle physiology. The result is a higher likelihood of arrhythmias, myocardial ischaemia, or overt heart failure.

Unfortunately, the transition period is marked by significant gaps in the management of these risk factors. Paediatric cardiologists typically focus on congenital defect surveillance and surgical outcomes, often without incorporating routine screening for metabolic or cardiovascular risk.

When patients transfer to adult cardiology services, clinicians may find themselves with incomplete information about earlier care, underestimating the need for early prevention, particularly when time and resources are constrained in busy outpatient settings.

Additionally, many young adults with CHD grow up perceiving their condition as fundamentally different from “typical” adult heart disease. This may create a false sense of protection from acquired risks [11]. As a result, they may be unaware of the importance of modifiable risk factors and may not feel the same urgency to adopt healthy behaviours as their peers with acquired cardiovascular conditions.

To close these gaps, transition planning must include a structured cardiovascular risk assessment. Ideally, this should begin before the actual transfer of care, during late adolescence. At this stage, clinicians should collect a comprehensive baseline cardiometabolic profile, including blood pressure, lipid levels, fasting glucose or HbA1c, BMI, and waist circumference. Screening should then continue at defined intervals, depending on individual risk stratification.

Treatment and prevention strategies should draw on general adult cardiology guidelines but must be adapted to the unique physiology and vulnerabilities of ACHD patients.

Lifestyle interventions—including healthy diet, regular physical activity, weight control, and smoking cessation—should be core components of the transition phase. These messages must be repeated and reinforced throughout follow-up, helping patients develop a proactive mindset toward long-term cardiovascular health [49].

Multidisciplinary transition clinics, in which paediatric and adult cardiologists collaborate, offer a highly effective model to facilitate seamless care and prevent patients from falling into gaps between services, ensuring that preventive cardiology becomes an integral part of lifelong ACHD follow-up [16].

**Figure 1:**
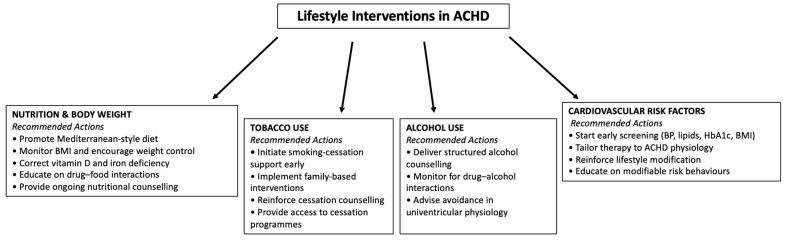
The figure provides an overview of the main recommended interventions across nutrition and body weight management, tobacco cessation, alcohol counselling, and cardiovascular risk-factor prevention.

A dedicated flowchart illustrates the core management actions across nutrition, tobacco use, alcohol consumption, and cardiovascular risk factors (Figure 1).

### 4.2. Physical Activity: Not Forbidden, but Tailored

Physical activity is universally recognised as a key pillar of cardiovascular health, offering benefits that extend well beyond the heart. It enhances functional capacity, improves endothelial function, supports emotional well-being, and plays a central role in the management of modifiable risk factors such as obesity, insulin resistance, and hypertension. However, in patients with ACHD, the relationship with exercise is more complex. It must be carefully calibrated to maximise benefit while minimising risk, particularly during the transition from paediatric to adult care, when the desire for independence and evolving identity may interfere in different ways.

For many adolescents with congenital heart disease, participation in physical activity—particularly sports—is more than just a health matter; it is deeply tied to social integration and a sense of normalcy. As they mature and seek greater autonomy, young patients often aspire to engage in the same athletic pursuits as their peers. Unfortunately, the medical system does not always provide the tools or clarity to safely support this goal. Many patients arrive in adult cardiology settings with vague or overly cautious instructions inherited from childhood (e.g., “avoid competitive sports”), but without clear, individualised guidance. At the same time, some adult cardiologists may feel less comfortable prescribing exercise regimens tailored to congenital anatomy and physiology, leading them to default to conservative restrictions.

This can create significant frustration for patients and families and may contribute to physical inactivity, loss of fitness, social withdrawal, or even disengagement from medical follow-up.

To avoid these pitfalls, best practice in ACHD care calls for a proactive and personalised approach to physical activity, beginning well before transfer to adult care. Functional assessment should be part of standard transition planning, often starting in mid-to-late adolescence. Objective evaluations, such as cardiopulmonary exercise testing (CPET) or the 6 min walk test (6MWT), offer valuable insights into a patient’s exercise capacity, chronotropic competence, and potential limitations. These tests allow for risk stratification and the development of tailored exercise prescriptions, including recommended heart rate ranges, intensity zones, session duration, and appropriate frequency.

In more complex cases, or when uncertainty arises, collaboration with sports cardiologists or exercise physiologists experienced in adult congenital heart disease can be instrumental in ensuring safe participation. These specialists can help define not only which activities are appropriate but also how to modify them when needed [50].

Physical activity counselling should be integrated into the transition phase, with a focus on empowering young patients to understand and manage their own activity levels. Teaching them how to self-monitor for symptoms (such as palpitations, dizziness, or undue fatigue), interpret heart rate data, and recognise warning signs can increase their confidence and safety. Regular follow-up and periodic reassessment are also essential, as a patient’s functional capacity, arrhythmia burden, or ventricular performance may evolve over time, necessitating adjustments to their exercise plan.

The 2020 ESC Guidelines for ACHD [16] recognise physical activity not merely as a lifestyle choice but as a clinical tool that contributes meaningfully to prognosis, quality of life, and cardiovascular resilience. When planned and monitored appropriately, physical activity becomes a powerful means to promote autonomy, reinforce self-efficacy, and support lifelong health—all foundational to a successful transition into adulthood for patients living with CHD.

### 4.3. Infective Endocarditis Prevention: Education on Daily Habits

Among the complications that may affect the lives of patients with CHD, one of the most concerning is infective endocarditis (IE), which carries substantial morbidity and mortality [51,52,53].

Rigorous cutaneous and dental hygiene are crucial to prevent IE. Nail-biting has been reported in about one-third of CHD patients, particularly among younger, obese individuals, those with complex CHD, and patients with learning disabilities. By causing trauma to the gingival margin and oral mucosa, nail-biting provides a favourable environment for bacterial colonisation and infection. Therefore, addressing the underlying causes and treatment of nail-biting is essential [54].

Several studies have demonstrated poorer oral health in children with CHD compared with healthy controls, mainly due to inadequate disease awareness and limited dental knowledge within their families. Thus, education of parents and children should begin at an early stage and should be supported by close interdisciplinary collaboration between paediatric cardiologists and paediatric dentists. In addition, patients should be encouraged to see a dental specialist before any surgical or percutaneous procedure to eliminate potential infection sources [55,56,57].

Adolescents with CHD often wish to lead a “normal” life, and the desire for body modification through piercing and tattooing is part of this pursuit. The risk of tattooing-related IE is considered low; however, cases tend to involve congenital lesions and often require surgical repair in addition to intravenous antibiotics [58].

Body-piercing-related IE has been described in several case reports and reviews, with Staphylococcus aureus recognised as the most common pathogen involved [59,60,61].

Staphylococcus aureus IE, in particular, carries significant morbidity and mortality, with multisystem complications and/or death occurring in one-third of patients [62].

**Figure 2:**
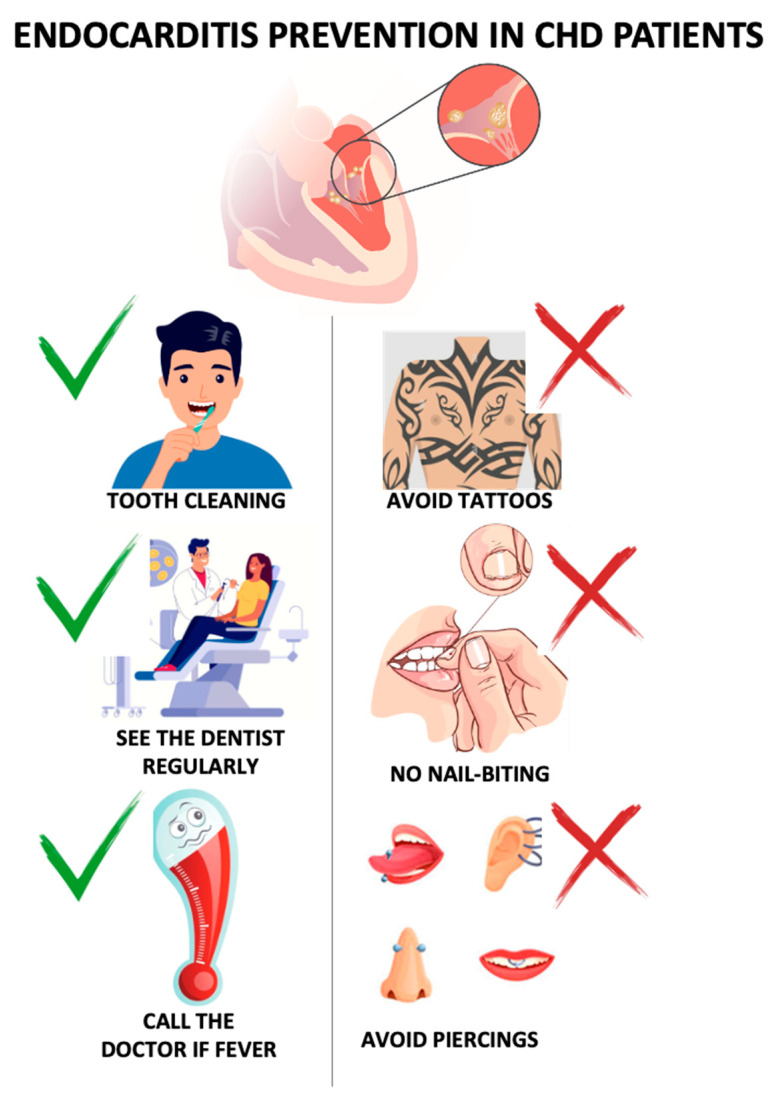
Measurements to prevent infective endocarditis in CHD. CHD = congenital heart disease.

Patients should also be encouraged to carefully monitor for signs of infection, contact their doctor in any case of unexplained fever, and avoid self-prescribing antibiotics (Figure 2).

Antibiotic prophylaxis (AP) is used to prevent IE in high-risk patients undergoing invasive dental procedures, but it may also be recommended for certain non-dental invasive procedures. Although AP reduces the risk of IE, it may promote the emergence of resistant microorganisms, thereby decreasing the efficacy and reducing the number of antibiotics available for the treatment of IE. Therefore, the use of AP should be carefully considered and tailored to the individual patient’s risk profile.

Recommendations on AP, patients at risk, and the types of procedures requiring AP are summarised in Table 1 [63,64].

In conclusion, education of young CHD patients and their families is crucial for the prevention of IE, with emphasis on maintaining strict dental and cutaneous hygiene, avoiding piercing and tattooing, and attending regular check-ups with a dental specialist.

### 4.4. Device Management

Cardiac implantable electronic devices (CIEDs) represent a unique challenge in CHD due to anatomical complexity, technical difficulties in smaller patients, and lifelong management issues, with a significantly higher rate of complications compared with non-CHD patients [65,66,67].

CIEDs considerably affect psychosocial functioning and quality of life (QoL), especially implantable cardioverter-defibrillators (ICDs) [68,69,70,71]. Even milder defects, when combined with a CIED, can have an impact comparable to that of complex CHD [70].

Adequate counselling for patients and families at the time of implantation is paramount to improve outcomes, as underlined by the recent PACES consensus, and may promote future acceptance of the device [65].

Shared decision-making is particularly important in “grey zones” of CIED indications, especially for primary prevention of sudden cardiac death (SCD), where validated and specific risk factors are lacking for most CHDs [65,67].

Owing to their high prevalence, active screening and management of psychological and social problems are essential components of lifelong follow-up, particularly during the vulnerable transition period [65,68,70,71].

CIEDs can affect various areas of psychosocial functioning—such as self-perception, anxiety and depressive disorders, and sexual functioning—and addressing the specific underlying mechanisms of QoL impairment is a key step [68,69,70,71,72].

Cosmetic outcomes may also be relevant for some patients, particularly young women, and should be considered during procedural planning [73].

Sport participation is not an absolute contraindication, but it may be arrhythmogenic and can cause CIED damage, particularly during intense or contact recreational exercise and competitive sports [65,74,75].

On the other hand, beyond its psychosocial impact, tailored exercise prescription significantly improves prognosis in CHD [74,76]—especially in specific subsets such as Fontan patients [77,78].

Therefore, exercise prescription must be carefully discussed with patients, involving school and community figures such as coaches, particularly in the case of ICDs [65].

Personalised device programming is recommended, including individualised upper sensor and tracking rates in pacemakers to ensure chronotropic competence, and high-rate cut-off and long detection duration to reduce unnecessary shocks in ICDs [74,79,80].

Telemedicine—well accepted by young patients—can facilitate dedicated visits addressing psychosocial issues. Furthermore, remote device monitoring may allow device checks and programming, reducing the burden of frequent in-person visits and enhancing compliance with follow-up at tertiary CHD centres, particularly for patients living far away [65,80].

Optimal management and preventive measures, together with proper patient education, are paramount to reduce complications. Common CIED-related complications include infections, inappropriate shocks, lead failure, venous obstruction, and thromboembolism [65,67].

The risk of CIED infection is substantially higher in the CHD population, mainly due to younger age at implantation, underlying CHD, and intracardiac prosthetic material [81,82].

Lead extraction is generally indicated in infected or malfunctioning systems and is associated with a non-negligible risk of major complications, especially in younger patients [81,83].

Beyond the general strategies for IE prevention previously described, specific prevention measures should be adopted. Adequate pre-, peri-, and post-procedural strategies—such as appropriate management of antithrombotic therapy, antibiotic prophylaxis, topical S. aureus decolonisation, hair removal with electric clippers, and proper wound-care instructions—are essential [81].

Inappropriate shocks are more frequent in CHD patients due to a higher prevalence of sinus tachycardia, supraventricular tachycardias, T-wave oversensing, and lead failure [78], and may have a deleterious psychological impact [68,71].

Individualised device programming optimisation, aggressive treatment of supraventricular tachycardias, and prompt psychological support are essential [65,67,71].

Epicardial pacing avoids issues related to transvenous leads and is commonly adopted in smaller children, but it is associated with higher rates of lead failure and the need for surgical intervention [65,66].

Subcutaneous ICDs (S-ICDs), and, based on preliminary evidence, leadless pacemakers, represent attractive options to reduce CIED infections and intravascular or lead-related complications [77,84,85,86].

S-ICDs have shown good results and safety in the CHD population, with no increased risk of inappropriate shocks compared with transvenous devices, especially with newer algorithms and appropriate patient selection [85,86].

S-ICDs may be particularly suitable for patients with no pacing requirement, high infection risk, or challenging vascular access [67,81].

In conclusion, education on living with a CIED and device-specific counselling by trained staff should be incorporated into transition programmes. Implementation of preventive strategies against CIED-related complications and active psychosocial monitoring and intervention are essential to optimise lifelong care in CHD patients.

In this context, it is important to note that the relevance of autonomic assessment has been demonstrated in other chronic conditions. For example, Matusik et al. showed that standardised reflex tests, including heart rate variability, Valsalva manoeuvre, and orthostatic blood pressure response, can reliably identify subclinical autonomic dysfunction and correlate with disease severity. Although these data derive from systemic lupus erythematosus, they highlight how autonomic cardiovascular evaluation may provide clinically meaningful insights beyond traditional assessments. Applying similar approaches to CHD could help characterise autonomic imbalance, refine arrhythmic risk stratification, and support exercise tolerance evaluation, especially in patients with complex physiology or overlapping symptoms [87].

### 4.5. Pregnancy Desire and High-Risk Cardiac Conditions in CHD Patients

Pregnancy desire among women with CHD represents a complex and deeply personal issue spanning medical, psychological, and social dimensions. Advances in paediatric cardiology and surgery have markedly improved survival, allowing many women with CHD to reach reproductive age and consider pregnancy [88,89]. However, coexisting high-risk cardiac conditions introduce substantial challenges regarding maternal safety, foetal outcomes, and long-term quality of life, requiring a careful balance between reproductive aspirations and potential cardiovascular risks [90]. Women with CHD often experience ambivalence: the natural desire for motherhood may be tempered by fears of decompensation, arrhythmias, thromboembolic events, or sudden cardiac death [91], while social expectations and concerns about transmitting congenital defects further affect emotional well-being. High-risk conditions such as Eisenmenger syndrome, severe pulmonary hypertension, or systemic ventricular dysfunction carry extremely high maternal morbidity and mortality, frequently making pregnancy contraindicated [89,92]. Nonetheless, some women continue to express pregnancy desire, sometimes underestimating risks or lacking comprehensive counselling [93].

Preconception counselling is pivotal for informed decision-making. Multidisciplinary “pregnancy heart teams” involving cardiologists, obstetricians, anaesthesiologists, and psychologists optimise cardiac status, assess individual risk, and discuss alternatives such as assisted reproduction or adoption [88,94]. Risk stratification follows the modified WHO (mWHO) classification, which categorises maternal cardiovascular risk from Class I (no increased risk) to Class IV (pregnancy contraindicated). Women with mild lesions commonly fall into lower mWHO classes, whereas patients with Eisenmenger syndrome or severe ventricular dysfunction fall into mWHO Class IV and should be strongly discouraged from pregnancy [89,92]. For intermediate-risk women (mWHO II–III), transition and pregnancy planning require tailored management, regular preconception evaluation, and clear communication about the expected incidence of adverse outcomes. Validated risk-prediction tools such as CARPREG II and ZAHARA can support counselling by estimating risks including heart failure, arrhythmias, hospitalisation, or neonatal complications, and may help guide monitoring intensity during pregnancy and postpartum follow-up.

For those proceeding with pregnancy, shared decision-making, close surveillance, individualised pharmacologic management, and delivery planning in specialised centres significantly improve outcomes [90]. This is particularly important for women with mWHO > II, who require strict multidisciplinary follow-up to minimise complications [95]. Beyond clinical risk assessment, psychosocial support remains fundamental: feelings of guilt or frustration may arise when pregnancy is discouraged, underscoring the need for empathetic communication and psychological assistance [91,93]. Advances in contraception and personalised preconception planning further empower women to make safer reproductive choices [89,94]. Ultimately, balancing hope and safety requires a compassionate, multidisciplinary approach that recognises both the medical complexity and the human dimension of reproductive decision-making in women with CHD [88,90,92].

### 4.6. Sexual Health, Contraception, and Intimate Relationships

Sexual health represents a key but frequently overlooked component of care for adolescents and adults with CHD. Many patients report uncertainties regarding the safety of sexual activity, potential cardiac risks, hereditary transmission of congenital defects, and appropriate contraceptive choices. Studies show that concerns about symptoms during exertion, arrhythmias, body image issues, and psychosocial stressors may negatively affect sexual functioning and intimate relationships in this population.

Sexual activity generally corresponds to mild–moderate physical exertion, and for most patients with CHD, it is considered safe when tailored to individual haemodynamic status. Nonetheless, patients with severe ventricular dysfunction, cyanosis, pulmonary hypertension, or arrhythmia susceptibility may require stricter counselling. Contraceptive choice is particularly important, as oestrogen-containing contraceptives may pose thrombotic risks in women with cyanotic heart disease, mechanical valves, or Fontan circulation, while progestin-only or non-hormonal methods may be preferable in these settings.

Reproductive counselling should also address concerns regarding pregnancy planning, teratogenic medications, and genetic counselling when appropriate. Education should be age-appropriate, confidential, and ideally initiated during transition to reduce misinformation and improve autonomy. Psychological support may be beneficial for patients experiencing anxiety, low self-esteem, or disturbances in sexual relationships [95].

## 5. Systemic Barriers to Implementing Standardised Transition Programmes

Despite broad agreement on the need for structured transition pathways, the implementation of standardised programmes faces substantial systemic barriers. First, many healthcare systems lack dedicated resources, including protected time, trained transition coordinators, and integrated psychological services, which are essential for sustaining multidisciplinary care. Organisational fragmentation between paediatric and adult services—often operating under different hospital networks, funding models, and electronic record systems—further undermines continuity and prevents the development of unified protocols. In addition, the availability of specialised ACHD centres remains uneven across regions, creating geographical disparities in access to expert care. Primary care engagement is limited, as many general practitioners report insufficient knowledge of CHD complexity and are unaware of local adult congenital services. Beyond healthcare structures, schools and employers seldom receive adequate training or guidance on the specific needs of adolescents with CHD, reducing the effectiveness of community-based support. Finally, variability in national health policies, reimbursement mechanisms, and legal frameworks contributes to inconsistent adoption of transition models across Europe. Addressing these systemic constraints is essential to translate evidence into practice and ensure equitable, high-quality transition care for all patients with CHD.

## 6. Practical Recommendations for Transition in CHD

To support clinicians in daily practice, key recommendations can be summarised as follows. Transition should begin early in adolescence, with structured, developmentally appropriate education focusing on disease knowledge, self-management, lifestyle behaviours, and reproductive health. Families require guidance to progressively promote autonomy while maintaining emotional support. General practitioners should be actively involved through shared-care pathways, ensuring continuity after transfer and facilitating early detection of comorbidities. Schools and employers should be integrated into transition planning to address educational challenges, promote social inclusion, and support gradual adaptation to work environments. Preventive measures—including strict dental and cutaneous hygiene, appropriate antibiotic prophylaxis for high-risk patients, and clear counselling regarding tattooing and piercing—are essential to reduce the risk of infective endocarditis. Physical activity should be individually prescribed based on functional assessment, with clear instructions on safe exercise thresholds and warning symptoms. In patients with CIEDs, tailored device programming, education on shock management, and remote monitoring are central to reducing complications. Finally, structured psychological assessment and support should be integrated throughout transition, particularly for patients with anxiety, depression, or neurocognitive impairments. Collectively, these recommendations provide a practical framework to standardise transition care and enhance long-term outcomes in adolescents and adults with CHD. Table 2 summarises the main practical recommendations to support structured, multidisciplinary transition care in patients with congenital heart disease.

## 7. Conclusions: A Call for Action

Transition in CHD must evolve from an administrative referral to a proactive continuum of care. Every ACHD centre should implement structured pathways that begin early in adolescence and extend across the medical, psychological, educational, and social domains of young patients’ lives. Education must be personalised, repetitive, and developmentally appropriate—not limited to disease knowledge but expanded to lifestyle, reproductive counselling, self-management, and psychosocial support. The adult CHD population is growing in number and complexity, and failure to anticipate their evolving risks means losing decades of progress earned in childhood. Transition is therefore not a transfer; it is a form of prevention in disguise.

Although most available evidence and models of care originate from Europe and other high-income settings, the vast majority of patients with congenital heart disease worldwide are born and treated in low- and middle-income countries, where resources, specialist centres, and ACHD programmes are limited. In these contexts, achieving the “minimum core elements” of transition requires a pragmatic, scalable approach prioritising essential components: structured education on the heart condition, early engagement of families, clear communication pathways with primary care, and the establishment of referral networks to regional centres when advanced expertise is needed. Even simplified models—such as nurse-led educational sessions, community-based follow-up, and telemedicine support—can substantially improve continuity of care where cardiology resources are scarce.

For extremely high-risk patients, including those with Eisenmenger syndrome or a failing Fontan circulation, the transition period represents a critical window to address long-term prognosis, realistic expectations for adult life, and when appropriate, discussions surrounding end-of-life planning. These conversations must be approached with sensitivity, cultural awareness, and multidisciplinary support, ensuring that patients and families are fully informed while preserving dignity, autonomy, and hope.

A global call for action is therefore necessary: transition should be recognised as an essential, lifesaving component of lifelong CHD management, adaptable to different healthcare systems and responsive to the specific needs of vulnerable patient groups. Only by investing in structured, inclusive, and equitable transition models can we ensure that patients not only survive into adulthood but are empowered to truly thrive.

## Figures and Tables

**Table 1:** Recommendation on antibiotic prophylaxis in congenital heart disease (adapted from 2023 ESC Guidelines for the management of endocarditis).

Antibiotic Prophylaxis in Congenital Heart Disease				
**Patient Category**	**Procedures Requiring Antibiotic Prophylaxis**	**Recommended Antibiotics** *(single dose, 30–60 min before procedure)*	**Procedures *Not* Requiring Prophylaxis**	**Notes**
**High-Risk Patients**	**High-risk dental procedures** involving manipulation of gingival tissue, periapical region, or perforation of oral mucosa	**First-line (oral):** *Amoxicillin 2 g PO* (adults) *50 mg/kg PO* (children) **If unable to take oral meds:** *Ampicillin 2 g IV/IM* (adults) *50 mg/kg IV/IM* (children) **Penicillin allergy:** *Clindamycin 600 mg PO/IV* (adults) *20 mg/kg PO/IV* (children) *Azithromycin or clarithromycin 500 mg PO* (adults) *15 mg/kg PO* (children)	- Routine skin procedures (including tattooing, piercing) - Endoscopic procedures (GI/bronchoscopy without incision) - Colonoscopy - **Ear, nose, and throat procedures** without incision - Dialysis, transfusion - Bone marrow puncture	Prophylaxis is **only** recommended for dental procedures. All other procedures require **aseptic technique only**.
**Intermediate-Risk Patients**	**No routine indication for prophylaxis**	—	Same as above	Prophylaxis may be considered only in exceptional situations (e.g., infected or contaminated surgical fields).
**General Population (Low Risk)**	None	—	All procedures	No indication for prophylaxis.

*PO* = per os; *IM* = intramuscolar; *IV* = intravenous.

**Table 2:** Key recommendations for CHD transition care.

Category	Key Recommendations
**Patient & Family Education**	Start transition education early; provide age-appropriate CHD information; promote autonomy while guiding families.
**General Practitioners & Shared Care**	Involve general practitioners to ensure continuity; provide clear clinical summaries; monitor cardiovascular risk factors.
**School, Social & Work Support**	Use individualised education plans; address bullying; support workplace adaptations.
**Psychological & Neurocognitive Support**	Screen for anxiety/depression; include psychological care; use visual tools for cognitive deficits.
**Infective Endocarditis Prevention**	Reinforce dental/cutaneous hygiene; avoid high-risk practices; apply guideline-based prophylaxis.
**Nutrition & Lifestyle**	Promote healthy diet and weight; prevent smoking/alcohol misuse; monitor deficiencies and interactions.
**Physical Activity**	Perform Cardiopulmonary Exercise Testing / 6-Minute Walk Test; tailor exercise prescriptions; educate on warning symptoms.
**CIED Management**	Provide device counselling; optimise programming; use remote monitoring; prevent infections.
**System-Level Considerations**	Implement structured pathways; improve paediatric–adult communication; expand access to ACHD centres.

## Data Availability

The original contributions presented in this study are included in the article. Further inquiries can be directed to the corresponding author.
